# Mothers’ experiences with mHealth intervention for postnatal care utilisation in Nigeria: a qualitative study

**DOI:** 10.1186/s12884-022-05177-x

**Published:** 2022-11-16

**Authors:** Aanuoluwapo Omobolanle Olajubu, Boluwaji Reuben Fajemilehin, Temitope Oluwafemi Olajubu

**Affiliations:** 1 Department of Nursing Science, College of Health Sciences, Obafemi Awolowo University, Osun State, Ile-Ife, 220005 Nigeria; 2 Department of Family Medicine, Obafemi Awolowo University Teaching Hospitals Complex, Ile-Ife, Nigeria

**Keywords:** Postnatal, Experience, Mother, mHealth, Neonate, Childbirth, Nigeria

## Abstract

**Background:**

The postnatal period implies a crucial and delicate time for both the mother and the newborn. There is a growing body of evidence that is increasingly pointing to mHealth interventions as a potential tool for improved utilisation of maternal and child health services, including postnatal care. This can promote the health of mother and baby during this delicate period. However, the success of the interventions must be explored to validate their usefulness and reliability. Hence, this study explored the experiences of postpartum women on the usefulness of the mHealth intervention (postnatal care assistant) they received.

**Methods:**

Twenty women, who were involved in mHealth intervention were interviewed using a semi-structured interview guide. They were recruited from the intervention group of a quasi-experimental study that evaluated the effect of a mHealth intervention on the uptake of postnatal care services. Thematic analysis of data was done using NVivo software version 10.

**Results:**

Five major themes emerged from data shared by the participants. They are general feelings about the messages, benefits derived from the messages about pregnancy and hospital delivery, increased knowledge about baby care, facilitation of PNC utilisation and involvement of significant others in decision making. They affirmed that the information and reminder messages gave them the impetus to utilise postnatal care services.

**Conclusion:**

Mothers reported that mHealth intervention provided immense support and assistance during pregnancy and the reminder messages encouraged them to utilise postnatal care services. This study suggests that improved education and reminder messages via mobile phones are needed during pregnancy and after childbirth to promote mother and child health through the utilization of postnatal care services, and efforts to put this approach to action should be pursued.

## Introduction

The time frame that begins immediately after a baby is born and lasting up to six weeks (42 days) is known as the postnatal period [1]. It is a critical and delicate time for both mothers and newborns because the majority of postnatal maternal deaths occur in the first week following birth [2–4]. It is one of the most susceptible times in a woman’s reproductive life cycle [3, 5], and the absence of appropriate and timely postnatal care during this period could lead to significant morbidity and mortality for both mother and child [3, 6].

Maternal mortality is still unsatisfactorily high especially in low- and middle-income countries (LMIC) [7], and it has been reported that a greater percentage of these mortalities occur during the postpartum period [2–4]. According to the recommendation on postnatal care (PNC) services by the World Health Organisation (WHO), mother and child should have at least four postnatal contacts (the first 24 hours after birth, 48–72 hours, 7–14 days, and 6 weeks after birth) with healthcare providers within the first six-week postpartum period [8]. However, studies have documented that the utilisation of postnatal care, which has a high prospect of reducing maternal morbidity and mortality, is still suboptimal in various parts of the world, especially in LMICs such as Nigeria [4, 9–11].

Globally, the utilisation of mobile technology to support the achievement of health goals can change the approach of health service delivery [12, 13]. Studies have shown that the deployment of mobile health (mHealth) intervention has the potential of improving the uptake of postnatal care services [14, 15]. However, the effectiveness of such measure is predicated, among other things, on its acceptance by the intended end users, that is, the mothers. The experience of the mothers with regard to ease of use, contextual relevance to their situation, and missing elements will largely influence the success of such interventions.

Studies have reported the experiences of women in developed countries regarding mHealth intervention targeted at postnatal care [16, 17]. However, while there has been a number of mHealth related studies in LMIC [18–20], there is paucity of data on the experiences of the end-users. In Nigeria, to our knowledge, there is no study that explored women’s experience with PNC-related mHealth interventions.

This study, therefore explored the experiences of women with the mHealth intervention they received during pregnancy and in the postpartum period.

## Conceptual framework

Major theories and conceptual approaches have influenced research endeavours on utilisation of healthcare services over time. This study was guided by the framework of Andersen’s behavioural model [21] which describes the various concepts and interrelated factors that are associated with health service utilization and which consequently impact on health outcomes.

**Fig. 1:**
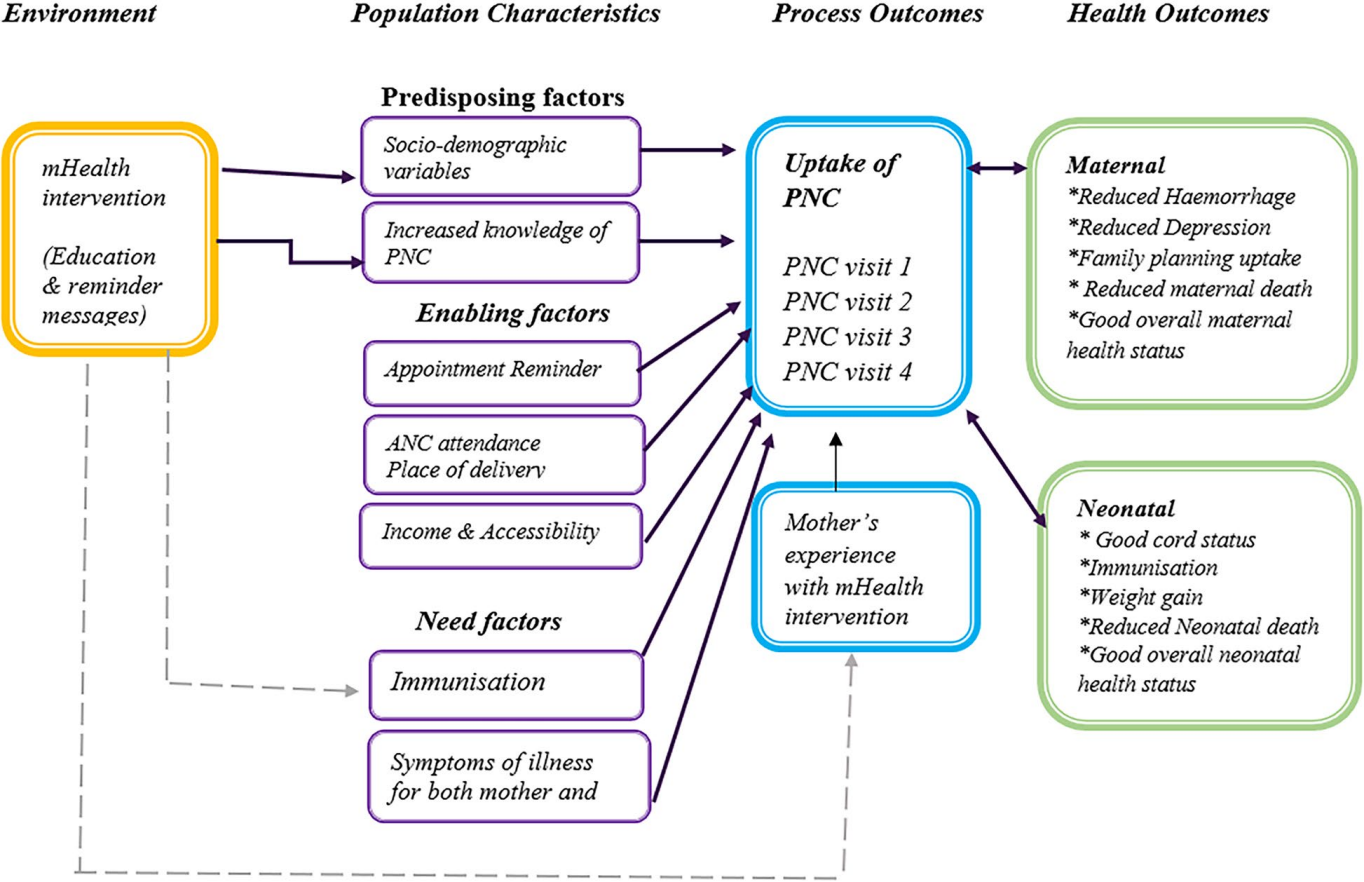
Conceptual model for experience of mothers on uptake of postnatal care services, adopted from andersen, 1995

The first broad concept espoused in the model is environmental factors which comprise of the healthcare delivery system and other factors external to the individual such as; various policy initiatives, public health education, health insurance schemes, etc. aimed at driving the distal factors towards increased service utilization and improved outcomes. Population characteristics, which is the second concept, include three characteristics: predisposing factors, enabling factors, and need factors. Examples of these include the client’s sociodemographic characteristics, level of health literacy and knowledge, socio-economic status, among others. One of the outcome measures envisioned by the model is consumer satisfaction (measured through the experiences of participants in this study), not only with the healthcare services received, but also with the external factors introduced proximally (Fig. 1).

This article reports the experience of mothers with mHealth educational and reminder messages introduced as part of a PNC mHealth intervention project. The project utilized mHealth intervention as an external factor aimed at influencing specific population characteristics such as predisposing (increased knowledge about postnatal care practices and services) and enabling factors (postnatal clinic appointment reminder). The goal was to stimulate positive health behaviours (i.e. healthy personal postnatal care practices and an increase in the uptake of postnatal care services) which will lead to positive health outcomes (i.e. improved maternal and neonatal health outcomes) (Fig. 1).

It is expected that utilization of the PNC services will also be impacted by the participants’ experience with the mHealth intervention. This informed the qualitative exploration of their experience and perception – regarding the usefulness of the mHealth program and its influence on their postnatal health behaviour and outcomes – as reported in this article.

## Methods

### Setting and selection of participants

The study used a descriptive qualitative research design also known as qualitative description to explore the experiences of mothers on the usefulness of mHealth intervention for postnatal care attendance. The term qualitative description is used in qualitative research to describe studies that are descriptive in nature, especially those examining phenomena related to health care and nursing [22]. It was adopted in this study to gain insight into the experiences of mothers on the mHealth intervention they received during pregnancy and after childbirth. Six Local Government Areas (LGAs) in Osun State, Nigeria, were randomly chosen for the study and divided into control and intervention groups. From the control (eight) and intervention (nine) groups, 17 primary health care (PHC) facilities that met the eligibility criteria as outlined in a prior article [23] were selected. Participants in this study were purposively recruited from the intervention group of a quasi-experimental study which evaluated the impact of mHealth intervention on the utilisation of postnatal care services among women in Osun State [23]. Mothers who registered for antenatal clinic in the intervention group, who were literate, possessed and able to operate simple functional mobile phones were included in the study while those who had any underlying medical conditions were excluded. Mothers who attended the sixth week postnatal clinic were purposively approached to share their experiences with the mHealth intervention received. Two participants who gave consent were interviewed in each of the nine healthcare facilities while two other participants were interviewed in the facility with the highest flow of clients making a total of 20 participants.

### Intervention

The mHealth intervention (postnatal care assistant) entailed an automated short messaging service (SMS) which provided educational messages relevant to maternal health and postnatal care attendance reminders. The intervention began at the 35th week of gestation and lasted till 6 weeks postpartum. During this period, each woman received both the educational and reminder text messages, based on their gestational ages and preferred languages. Healthy prenatal lifestyle habits, clinical care indications, the recommended number and timing of postnatal care visits, among other things, are some of the subjects covered. Further details of the mHealth intervention provided has been described in another article [23].

### Data collection procedure

After the intervention, women involved in the intervention were asked to participate in an interview with the intention of gaining understanding of, and documenting their overall experiences with the mHealth intervention. They were contacted for interviews in the selected healthcare facilities used for intervention; those who gave consent to participate were interviewed and data saturation was achieved with the 18th participant. However, two additional interviews were conducted to ensure that there was no new information. The interviews were conducted by the authors, with each lasting for an average of 35 minutes. Each interview, conducted in each participant’s preferred location and language (English or Yoruba), was audio recorded. Participants’ consents were sought, both in verbal and written forms and they were assured of confidentiality and privacy.

An interview guide comprising four sections; introduction, questions, conclusion and socio-demographic data was developed to explore the experiences of mothers and the usefulness of the intervention they received during pregnancy and after childbirth. The instrument was pretested with an interview in order to identify and implement useful modifications to the questions before actual field work.

Participants were asked to share their personal experiences about the postnatal care messages and reminders they received. They were also encouraged to be as objective as possible. Interviews were conducted between January and March 2018.

### Data analysis

The data from in-depth interviews were analysed using a thematic approach. Suggestions from Nowell et al. [24] on methodical steps were adopted in carrying out the thematic analysis. The interviews were transcribed verbatim from the audio recordings, and the transcripts were painstakingly reviewed. The transcripts of interviews conducted in Yoruba language were translated to English language before coding. All the transcripts were repeatedly read in order to search for codes and patterns. This was necessary because of the need to engage in appropriate meaning construction.

Two authors (AOO and TOO) worked independently on the review of the transcripts and the generation of initial codes (using colour coding). The codes were compared for uniformity and where there was any difference, this was resolved through consensus.

The documented codes were collated, sorted, and themes were generated inductively. Codes were compared and similar ones were grouped into subthemes. Following that, subthemes were compared and grouped into the main themes. The various themes and subthemes were extensively discussed during several meetings between the two authors to achieve confirmability and objectivity in data analysis.

Before deciding on the central themes, the authors compared and contrasted the themes. Subsequently, the themes were reviewed to be sure they were representative of the raw data. Illustrative quotes from the participants’ narratives were selected to highlight central themes. The NVivo software version 10 was used to sort, organise, and store the data set.

### Qualitative rigour

The criteria for ensuring the trustworthiness of qualitative studies according to Cuba and Lincoln as described in Forero et al. [25] were used in this study. Participants were encouraged to be as objective as possible, and to support their statements with relevant examples. Follow up questions were also asked to further clarify the information that the participants provided. The data collection was given enough time, and data saturation was ensured. Member check was ensured by presenting the interview transcripts to some of the participants to confirm the accuracy of the descriptions given during the interview and  to ascertain that the transcripts accurately portrayed the participants’ viewpoints. A detailed record and description of the research methods and procedure were presented. A skilful transcriber carefully transcribed the audio recorded data for analysis and two expert qualitative nursing researchers reviewed the transcribed material to validate the identified themes and quotes. The degree of neutrality in the findings of this study was ensured by keeping a reflexive journal throughout the research process to keep notes and document information that were helpful and relevant during the study. Also, the researchers constantly self-reflected about their own biases, values and preferences in order to clarify and separate them from the data that the participants provided. These steps along with those described earlier under data analysis were used to ensure the credibility and trustworthiness of this study.

## Result

### Profile of the study participants

Twenty mothers who received the mHealth intervention (postnatal care assistance) were interviewed. Their ages ranged between 24 and 38 years, and all were married. Majority (75%) were Christians, about two-third (65%) had secondary school education and more than one-third (40%) were traders. Table 1 presents detailed sociodemographic characteristics of the participants.

**Table 1:** Sociodemographic Characteristics of Participants

Participants	Age (Years)	Education	Occupation	Religion
1	35	Secondary	Trader	Christianity
2	25	Secondary	Artisan	Islam
3	25	Primary	Trader	Christianity
4	36	Secondary	Business	Christianity
5	35	Tertiary	Civil Servant	Christianity
6	31	Secondary	Unemployed	Christianity
7	26	Tertiary	Business	Christianity
8	32	Primary	Artisan	Christianity
9	30	Secondary	Artisan	Christianity
10	28	Secondary	Trader	Islam
11	25	Secondary	Artisan	Christianity
12	29	Tertiary	Unemployed	Islam
13	29	Secondary	Trader	Christianity
14	38	Secondary	Trader	Christianity
15	24	Secondary	Artisan	Christianity
16	32	Secondary	Trader	Islam
17	37	Tertiary	Civil Servant	Christianity
18	29	Secondary	Business	Christianity
19	30	Secondary	Trader	Islam
20	32	Primary	Trader	Christianity

### Experiences of mothers on mHealth intervention

The findings revealed five major themes which emerged from participants’ narratives: general feelings about the messages, benefits derived from the messages about pregnancy and  hospital delivery, increased knowledge about baby care, facilitation of PNC utilisation and involvement of significant others in decision making (Table 2).

**Table 2:** Description of themes and subthemes

S/N	Themes	Sub-themes
1	General feelings about the messages	∙ Relevance and usefulness ∙ Challenges experienced
2.	Benefits derived from the messages about pregnancy and hospital delivery	∙ Information on late stage of pregnancy ∙ Knowledge about self-care ∙ Utilisation of hospital-based intrapartum care
3.	Increased knowledge about baby care	∙ Better infant care practices ∙ Breastfeeding ∙ Cord care ∙ Immunisation
4.	Facilitation of postnatal care utilisation.	∙ Knowledge of the recommended postnatal care services ∙ Reminder for utilisation of postnatal care services ∙ Factors influencing postnatal care utilisation
5.	Involvement of significant others in decision making	∙ Husband’s involvement ∙ Mother’s involvement

Participants described the encouragement they received from the messages and expressed how useful they were during the course of their pregnancies. They also highlighted the benefits of these messages to them as regards pregnancy and decisions to give birth in the hospital. The knowledge acquired from these messages in terms of breastfeeding, cord care and immunisation were emphasised. Furthermore, information about postpartum, as well as reminder messages were underscored as very important. Even though these experiences are being reported separately here, they were interrelated and interwoven within the context of the participants’ narratives.

### Theme 1: general feelings about the messages

Majority of the women described how elated and encouraged they were to have received the mHealth messages.

#### Relevance and usefulness

Most of the participants acknowledged that the messages were apt, relevant and very useful to them. Many of them said they learnt new things which were very practical, while others said the knowledge they had before were reinforced. The participants descriptions of the usefulness of the messages and sense of reassurance it gave are as follows:

Those text messages were very good; I was always happy any time they came in to my phone. Even though I knew about many of the information because this is my fourth child, they helped me to know more about the right things to do. They were very useful. (Participant 1).

Those messages were very good and useful, they actually prompted me to do a number of things differently and to take better care of this baby than I did with my first child. (Participant 9).

#### Challenges experienced

The challenges experienced by few participants in relation to the messages received during intervention were highlighted in this section. One of the women said she was unable to read the messages because the screen of her phone was bad and was only useful for voice calls:

Hmmmn, those messages …, I received them but could not read most of them. The phone screen got spoilt, I could only make or receive calls with it. I’m sorry. (Participant 15).

Another participant expressed that she had to give her phone to people to help explain some messages she can’t understand:

Any of the texts that I didn’t understand well, I gave it to someone to explain to me because I didn’t want to miss the useful information contained in the messages. (Participant 1).

### Theme 2: benefits derived from the messages about pregnancy and hospital delivery

#### Information on late stage of pregnancy

Most of the participants described how the text messages helped them to have better understanding of certain symptoms and challenges that are associated with the late stages of pregnancy. These include backaches, fatigue, mood swings, and stronger perception of the movement of the foetus which tends to be associated with some discomforts.

Participant 5 explained that this enhanced understanding helped her to be less anxious, calm and re-assured whenever she experienced some of these symptoms:

There was a particular time I was very irritable towards the end of the pregnancy period. Coincidentally, that was the exact period that one of the messages came in, yes …the message said it is normal to sometimes feel irritable, so I just laughed (laughing ….) and it calmed me down. It was quite interesting.

Participant 10 also affirmed as follows:

Hhmmm……I used to be very scared whenever the baby moves inside me in an unusual way or sometimes very vigorously but when I started receiving the messages, they contained information that explained so many things which were always reassuring to me.

#### Knowledge about self-care

Some of the participants claimed that increased knowledge about self-care during pregnancy was the key benefit they attributed to the information gleaned from the text messages they received. Two key aspects were identified to be of greatest impact; information on healthy diet during pregnancy and how to maintain proper balance between daily activities and adequate rest:

Those messages ehn…they encouraged me a lot. I learnt the kind of food to eat during pregnancy and how to maintain proper balance between the stress of daily activities and adequate rest. (Participant 3).

Another participant said:

The messages helped me so much in knowing how to take proper care of myself during pregnancy especially towards the last few weeks and how to prepare for labour and delivery. (Participant 7).

#### Utilisation of hospital-based intrapartum care

Some of the participants attributed their decision to give birth in a health facility to the lessons and encouragement obtained from the SMS messages they received during the intervention: The statement of participant 14 is as follows:

The messages helped me to understand and appreciate the importance of having my delivery in the healthcare facility. Before, I used to give birth in the church, but this time, it was at the clinic and the messages actually prompted me to do so.

Participant 3 expressed her gratitude for how the decision to give birth in a hospital as a result of the mHealth intervention messages saved her life and that of her baby. She revealed that she went through a difficult labour process as follows:

I went through a lot during my labour, it was a little difficult. The healthcare workers had to intervene in different ways. Thank God I came to deliver in the hospital, I don’t know what would have happened to me or the baby. I might not have used the hospital if not for the messages.

Participant 7, who was also persuaded by the educational messages to give birth in a hospital, described how she experienced postpartum bleeding, which could have had fatal consequences if she had given birth at home or under the care of a traditional birth attendant:

My last delivery was not in the hospital. The information in the text messages helped in encouraging my decision to use the hospital this time around. It turned out that I started bleeding after I gave birth which was promptly managed by the healthcare workers.

### Theme 3: increased knowledge about baby care

#### Better infant care practices

This theme was extensively discussed by majority of the participants. It accounts for participants’ experience of enriched capacity to take better care of their babies by the implementation of proper infant care practices learnt or reinforced through the educational text messages as follows:

The messages gave me more knowledge than I had before about caring for my baby. I discovered that I didn’t really take care of my first born in the right ways, no wonder he was frequently falling sick… but this particular baby has been very healthy which I believe is because of what I know now. (Participant 4).

The main aspects emphasised by overlapping subsets of the mothers include breastfeeding, cord-care, and immunisation.

#### Breastfeeding

According to most of the participants, the messages helped them further appreciate the importance of exclusive breastfeeding for up to 6 months. A participant said she never knew she was to exclude water:

I am the type that used to give water along with breastfeeding and from 3 months I give pap. I actually didn’t know I should not give water at all, but this time around, as encouraged by the messages, I’m trying to give breast milk only for 6 months and you can see my baby…, she’s looking very fine and healthy... (laughs). (Participant 14).

Furthermore, there were a number of other new things about the art of breastfeeding which a few of the participants acknowledged that they learnt. These include: signs that suggest that a baby is in a comfortable posture and suckling effectively (*Participant* 4); and the need for regular feeding, rather than wait for the baby to cry before feeding him/her (*Participant* 14).

The lesson on the relationship between mothers’ diet and the quality of the breast milk produced was also noted as quite informative:

I learnt about the importance of eating well in order to breastfeed well and the need to take food- like vegetables, fruits, beans etc. (Participant 11).

#### Cord care

A number of the participants explained how the mHealth messages helped them do away with their traditional cord care practices which involves the application of dry heat with a cloth heated up on a lantern. One of the participants stated:

The information was quite strange to me despite this baby being my fourth child, it was contrary to the age-long practice I have always heard and known of. I learnt about the proper way to take care of the cord for the first time from the messages sent to me. I however decided to implement the change because of the confidence I had in the trustworthiness of the information contained in the messages. (Participant 4).

#### Immunisation

While information about the importance and schedule of immunisation was not entirely new for most of the mothers, some of them, however, acknowledged that the messages reinforced their knowledge and positive attitude towards it.

Most of them further commented that they benefited immensely from the fact that the messages served as reminders for the next scheduled immunisation as explained by a participant:

Some of the most important of all the messages for me were the ones on immunisation…and especially as they also reminded me of the expected immunisation days*.* (Participant 2).

A participant emphasised how the messages helped to dispel a myth she had about immunisation. The misconception was reportedly due to a previous experience she had with her older child as expressed:

I lost interest in vaccination because of the experience with my previous baby, he always cries repeatedly every day after each dose of the injections. I decided not to take the vaccines for this baby but your messages convinced me again on the importance of immunisation. I changed my mind and have been taking him for immunisation. Interestingly, the baby has not actually been crying. (Participant 5).

### Theme 4: facilitation of postnatal care utilisation

Another experience that majority of the participants shared was in relation to the impact of the messages on their awareness, knowledge and utilisation of postnatal care services.

#### Knowledge of recommended postnatal care services

Participants unanimously avowed that their knowledge about PNC was immensely enriched with regard to its importance, the services to expect, and the number and timing of PNC visits.

Many of them opined that the information about routine postnatal check-up and the clinic day reminder messages were among the most important of the various text messages they received as illustrated by the following quotes:

All the messages were good and helpful, but for me, it was particularly interesting and important to learn that I was supposed to visit the clinic for postnatal check-up on certain days, I think on day 3, day 10 and at 6 weeks … I hope I’m correct. I was always reminded of my next visiting day and I always tried to follow the instructions*.* (Participant 2).

I love the messages on postnatal visits because they taught me the appropriate days to come for postnatal clinic. (Participant 12).

#### Reminder for utilisation of postnatal care services

Most of the participants underscored the role of the messages as reminders for clinic days as very useful. They responded positively to these messages by attending the clinics. With a few exceptions, all those who utilised the PNC services acknowledged that they would not have thought of doing so if not for the information and promptings occasioned by the messages as illustrated by the following quotes:

The messages informed my decision to attend all the postnatal clinics. Even if the healthcare workers have told me to come for check-up after birth, I would not have remembered to come or attach much importance to it if it had not been for the educational messages and consistent reminders. (Participant 7).

It was **t**he messages that really helped and prompted me to make use of the clinic, I don’t usually come back to the hospital after delivery even if I have any challenge, I prefer to go to the chemist shop. But this time around, I attended the postnatal clinic and also came to receive treatment at a time that I was sick. (Participant 13).

#### Factors influencing postnatal care utilisation

A few of the participants, volunteered information on factors that hindered them from implementing instructions from the text messages regarding postnatal care. The main reasons mentioned were financial limitation and being too busy to wait in the hospital, as described in the following quotes:

Even though I received the text messages about postnatal check-up and services, I could not go for two check-ups because I didn’t have enough money on me at the time. (Participant 16).

On the day I went for the first PNC check-up as recommended in the text messages, the clinic was too crowded, I had to spend a long time in the clinic. This discouraged me from going back subsequently because I am a very busy business person, there is a lot to do in addition to taking care of the child. (Participant 18).

### Theme 5: involvement of significant others in decision making

Few participants expressed the impact of the involvement of their significant others in following through with the instructions in the messages they received during the intervention.

#### Husband’s involvement

A participant discussed the involvement of her husband in ensuring the implementation of the information in the proper way to take care of the baby’s cord stump. She said:

My husband was also receiving same messages on his phone. He even loved them more than I do. When he saw the message on how to clean the baby’s cord with methylated spirit only, rather than the way we used to do it, he insisted that we should do it as instructed. (Participant 9).

#### Mother’s involvement

One of the participants also expressed a sentiment of giving birth under the supervision of a traditional birth attendant as suggested by her mother. She said:

Though I received the messages about the importance of having one’s labour and delivery in a healthcare facility, I eventually delivered in a mission house in view of my mother’s insistence…you know what I’m talking about. (Participant 20).

## Discussion

The objective of this study was to explore the experiences of women on the content and usefulness of the mHealth intervention they received. Five major themes emerged from participants’ narratives; general feelings about the messages, benefits derived from the messages about pregnancy and  hospital delivery, increased knowledge about baby care, facilitation of PNC utilisation and involvement of significant others in decision making.

The participants were excited and stimulated by the mHealth messages they received which gave them information about their pregnancy and child birth, and also reminded them of the postnatal care attendance. This is typical of pregnant women because they usually desire to receive information about their health and that of their babies. This corroborates a previous study where mothers confirmed their receptiveness and gratification toward using the mHealth application for postnatal education in Singapore [16].

None of the participants had any feeling of “being disturbed” or saw the text messages as constituting a nuisance. This denotes that the participants had genuine interest in receiving the mHealth messages to improve their knowledge, and to remind them of postnatal care appointments. This is also in congruence with a previous study where women demonstrated interest in using the application for future pregnancies, and recommending it to their friends who were expectant [16]. One of the participants, however, reported that she got the messages but could not read because her phone screen was bad. This might have hindered her from utilising the contents of messages sent to her.

Participants reported that the text messages received improved their knowledge of symptoms and challenges commonly associated with the late stages of pregnancy, which eventually led to less anxiety during their pregnancy. This was a good feedback from the participants because increased knowledge has a great likelihood of leading to change in behaviour. A similar finding was reported in Ethiopia where mother’s knowledge on postnatal danger signs was found to be significantly associated with postnatal care service utilisation [26].

Decisions to give birth at the healthcare facility was also reported by the participants as one of the aftermaths of the messages they received. This was very important since it has been shown that a significant association exists between giving birth in the hospital and utilisation of postnatal care services [26].

Almost all the participants reported improved ability in taking better care of their babies by the implementation of proper infant care practices learnt through the educational text messages they received. A number of the participants explained how the mHealth messages helped them do away with the traditional cord care practices involving the application of dry heat with a cloth heated up on a lantern. This was an indication that with improved mother’s knowledge on maternal and neonatal health, harmful traditional practices around pregnancy, childbirth and baby care can be eradicated.

Nearly all the mothers shared their experiences in relation to the impact of the messages on their awareness, knowledge and utilisation of postnatal care services. They reported that their knowledge about PNC was immensely enriched with regard to its importance, the services to expect, as well as the number and timing of PNC visits.

Many of them opined that the information about routine postnatal check-up and the clinic day reminder messages were among the most important of the various text messages they received. Majority of the women acknowledged that they would not have thought of utilising postnatal health care services, if not for the information and prompts they got from the messages. This is comparable to a previous study where text message reminders led to a reduction by half, of failure to attend postnatal clinic appointments [27], and also to other studies, where it was found that text messages increased the possibility of utilising maternal healthcare services [23, 28–31].

Despite the messages, one participant still could not attend PNC due to financial limitations. This is similar to the submission in a previous study, where mothers with low per capita income were less likely to fully utilise PNC services as a result of financial difficulty [32]. Moreover, a few of the mothers offered information on factors that hindered them from implementing the text messages on postnatal care. The main reasons they gave include financial limitation and being too busy to wait in the hospital. This is in congruence with findings from previous study where socioeconomic status influenced the use of PNC services [33] and being too busy with other family matters [26].

Another participant explained that her husband was involved in ensuring the implementation of instruction on the proper way to take care of their baby’s cord. This might be traced to the fact that messages were not only sent to mothers but also to their partners, in order to enhance women’s decision-making processes. This outcome was of great significance in a setting where fathers are key to the decision-making process of the family [14].

Despite the messages received, one of the participants still couldn’t resist her mother’s insistence to deliver in the care of traditional birth attendants which led her to give birth outside the healthcare facility. This is not unexpected because of the influence of significant others in decision making process in Africa setting, especially Nigeria.

## Strength and limitation

This is the first study in Nigeria to explore the experiences of women on technology-based educational and reminder messages for WHO’s recommended postnatal care services. The qualitative method provided an in-depth insight into the perspective of mothers on how mHealth intervention can be of assistance in taking care of their babies, as well as the utilisation of healthcare facilities in the course of pregnancy, childbirth, and even in the postpartum period. However, the perspectives of healthcare workers in the acceptance and utilisation of mHealth intervention for uptake of postnatal care are vital, and this was not captured in this study, hence, future studies are recommended in this regard.

## Conclusion

The mHealth intervention was well received by mothers. They conveyed their positive experiences regarding the usefulness of educational and reminder messages, including the fact that the mHealth intervention propelled their utilisation of postnatal care services. The findings suggest a potential role for mHealth intervention in improving PNC uptake. Hence, community health nurses and midwives can leverage on this prospect to ensure the optimal utilisation of postnatal care services. Also, public health policy makers and relevant stakeholders may need to explore the opportunities that mHealth intervention offers.

## Data Availability

The datasets used and analysed during the current study are available from the corresponding author on reasonable request.

## Abbreviations

LMIC: Low - and middle-income countries
mHealth: Mobile Health
PNC: Post Natal Care
PHC: Primary Health Care
SMS: Short Messaging Service
WHO: World Health Organisation

## References

[1] Finlayson K, Crossland N, Bonet M, Downe S (2020). What matters to women in the postnatal period: a meta-synthesis of qualitative studies. PLoS One [Internet].

[2] Mon AS, Phyu MK, Thinkhamrop W. Open Peer Review Discuss this article (0) Comments Utilization of full postnatal care services among rural Myanmar women and its determinants: a cross-sectional study. F1000Research. 2018;7. 10.12688/f1000research.15561.1 PMC6085599 30135735

[3] World Health Organization. WHO recommendations on postnatal care of the mother and newborn [Internet]. World Health Organization; 2014. Available from: http://apps.who.int/iris/handle/10665/97603. 24624481

[4] Wudineh KG, Nigusie AA, Gesese SS, Tesu AA, Beyene FY. Postnatal care service utilization and associated factors among women who gave birth in Debretabour town, North West Ethiopia: A community- based cross-sectional study 11 Medical and Health Sciences 1114 Paediatrics and Reproductive Medicine. BMC Preg Child. 2018;18(1). 10.1186/s12884-018-2138-x PMC6307219 30591039

[5] Kinuthia PM (2014). Factors affecting utilization of postnatal care services in Kenya. South Am J Public Heal [Internet].

[6] Sardi L, Idri A, Readman LM, Alami H, Bezad R, Fernández-Alemán JL (2020). Mobile health applications for postnatal care: Review and analysis of functionalities and technical features. Comput Methods Programs Biomed.

[7] World Health Organization. Trends in maternal mortality 2000 to 2017: estimates by WHO, UNICEF, UNFPA, World Bank Group and the United Nations Population Division. Geneva: World Health Organization; 2019. Licence: CC BY-NC-SA 3.0 IGO.

[8] Sakamoto JL, Carandang RR, Kharel M, Shibanuma A, Yarotskaya E, Basargina M (2022). Effects of mHealth on the psychosocial health of pregnant women and mothers: a systematic review. BMJ Open.

[9] Abebo TA, Tesfaye DJ (2018). Postnatal care utilization and associated factors among women of reproductive age Group in Halaba Kulito Town. Southern Ethiopia Arch Public Heal.

[10] Olajubu AO, Olowokere AE, Ogundipe MJ, Olajubu TO (2019). Predictors of Postnatal Care Services Utilization Among Women in Nigeria: A Facility-Based Study. J Nurs Scholarsh.

[11] Tsawe M, Moto A, Netshivhera T, Ralesego L, Nyathi C, Susuman AS (2015). Factors influencing the use of maternal healthcare services and childhood immunization in Swaziland. Int J Equity Health.

[12] Haluza D, Jungwirth D (2014). ICT and the future of health care: aspects of health promotion. Int. J. Med. Inform..

[13] Kay M, Santos J, Takane M. mHealth: New horizons for health through mobile technologies. World Heal Organ [Internet]. 2011;66–71. Available from: http://www.who.int/goe/publications/goe_mhealth_web.pdf

[14] Olajubu AO, Fajemilehin BR, Olajubu TO, Afolabi BS (2020). Effectiveness of a mobile health intervention on uptake of recommended postnatal care services in Nigeria. . Owolabi OO. PLoS One.

[15] Sondaal SFV, Browne JL, Amoakoh-Coleman M, Borgstein A, Miltenburg AS, Verwijs M (2016). Assessing the effect of mHealth interventions in improving maternal and neonatal Care in low-and Middle-Income Countries: a systematic review. PLoS One.

[16] Shorey S, Yang YY, Dennis CL (2018). A mobile health app-based postnatal educational program (home-but not alone): Descriptive qualitative study. J Med Internet Res.

[17] Sadural E, Riley KE, Zha P, Pacquiao D, Faust A (2022). Experiences with a postpartum mHealth intervention during the COVID-19 pandemic: key informant interviews among patients, health care providers, and stakeholders. JMIR Form Res.

[18] Kebede AS, Ajayi IOO, Arowojolu AO. Effect of enhanced reminders on postnatal clinic attendance in Addis Ababa, Ethiopia: a cluster randomized controlled trial. Glob health action. 2019;12(1). 10.1080/16549716.2019.1609297. 10.1080/16549716.2019.1609297 PMC6534243 31124401

[19] Shiferaw S, Spigt M, Tekie M, Abdullah M, Fantahun M, Dinant GJ (2016). The effects of a locally developed mHealth intervention on delivery and postnatal care utilization; a prospective controlled evaluation among health centres in Ethiopia. PLoS One.

[20] Prinja S, Nimesh R, Gupta A, Bahuguna P, Gupta M, Thakur JS (2017). Impact of m-health application used by community health volunteers on improving utilisation of maternal, new-born and child health care services in a rural area of Uttar Pradesh. India Trop Med Int Heal.

[21] Andersen RM (1995). Revisiting the behavioral model and access to medical care: does it matter?. J Health Soc Behav..

[22] Polit DF, Beck CT. Essentials of Nursing Research: Appraising Evidence for Nursing Practice - Denise F. Polit, Cheryl Tatano Beck - Google Books [Internet]. 2018 [cited 2022 Aug 5]. Available from: https://books.google.com.ng/books?hl=en&lr=&id=7GtP8VCw4BYC&oi=fnd&pg=PA258&dq=Polit,+D.+F.,+%26+Beck,+C.+T.+(2009).+Essentials+of+nursing+research:+Appraising+evidence+for+nursing+practice.+Lippincott+Williams+%26+Wilkins.&ots=kH-Fg4sG2_&sig=4Yh_-GWgZ-Uo.

[23] Olajubu AO, Fajemilehin BR, Olajubu TO, Afolabi BS (2020). Effectiveness of a mobile health intervention on uptake of recommended postnatal care services in Nigeria. Plos one.

[24] Nowell LS, Norris JM, White DE, Moules NJ. Thematic Analysis: Striving to Meet the Trustworthiness Criteria Available from: https://us.sagepub.com/en-us/nam/open-access-at-sage.

[25] Forero R, Nahidi S, De Costa J, Mohsin M, Fitzgerald G, Gibson N (2018). Application of four-dimension criteria to assess rigour of qualitative research in emergency medicine. BMC Health Serv Res.

[26] Belachew T, Taye A, Belachew T (2016). Postnatal care service utilization and associated factors among Mothersin Lemo Woreda, Ethiopia. J Women’s Heal Care.

[27] Adanikin AI, Awoleke JO, Adeyiolu A (2014). Role of reminder by text message in enhancing postnatal clinic attendance. Int J Gynecol Obstet.

[28] Fedha T. Impact of mobile telephone on maternal health service care: a case of Njoro division. Open J Prev Med. 2014;2014.

[29] Lau YK, Cassidy T, Hacking D, Brittain K, Haricharan HJ, Heap M (2014). Antenatal health promotion via short message service at a midwife obstetrics unit in South Africa: a mixed methods study. BMC Preg Child..

[30] Lund S, Nielsen BB, Hemed M, Boas IM, Said A, Said K (2014). Mobile phones improve antenatal care attendance in Zanzibar: a cluster randomized controlled trial. BMC Preg Child.

[31] Mushamiri I, Luo C, Iiams-Hauser C, Ben AY (2015). Evaluation of the impact of a mobile health system on adherence to antenatal and postnatal care and prevention of mother-to-child transmission of HIV programs in Kenya. BMC Pub Health.

[32] Mon AS, Phyu MK, Thinkhamrop W, Thinkhamrop B. Utilization of full postnatal care services among rural myanmar women and its determinants: A cross-sectional study. F1000Research. 2018;7. 10.12688/f1000research.15561.1 PMC6085599 30135735

[33] Mukonka PS, Mukwato PK, Kwaleyela CN, Mweemba O, Maimbolwa M (2018). Household factors associated with use of postnatal care services. Afr J Midwifery Womens Health.

